# Chemogenetic activation or inhibition of histaminergic neurons bidirectionally modulates recognition memory formation and retrieval in male and female mice

**DOI:** 10.1038/s41598-024-61998-0

**Published:** 2024-05-17

**Authors:** Alessia Costa, Eva Ducourneau, Lorenzo Curti, Alessio Masi, Guido Mannaioni, Lola Hardt, Essi F. Biyong, Mylène Potier, Patrizio Blandina, Pierre Trifilieff, Gustavo Provensi, Guillaume Ferreira, M. Beatrice Passani

**Affiliations:** 1 Department of Neuroscience, Psychology, Drug Research and Child Health, Pharmacology and Toxicology Unit, University of Florence, Florence, Italy; 2 INRAE, Bordeaux INP, Nutrition and Integrative Neurobiology, UMR 1286, University of Bordeaux, 33077 Bordeaux, France; 3 Department of Health Sciences, Clinical Pharmacology and Oncology Unit, University of Florence, Florence, Italy

**Keywords:** Neuroscience, Learning and memory

## Abstract

Several lines of evidence demonstrate that the brain histaminergic system is fundamental for cognitive processes and the expression of memories. Here, we investigated the effect of acute silencing or activation of histaminergic neurons in the hypothalamic tuberomamillary nucleus (TMN^HA^ neurons) in vivo in both sexes in an attempt to provide direct and causal evidence of the necessary role of these neurons in recognition memory formation and retrieval. To this end, we compared the performance of mice in two non-aversive and non-rewarded memory tests, the social and object recognition memory tasks, which are known to recruit different brain circuitries. To directly establish the impact of inactivation or activation of TMN^HA^ neurons, we examined the effect of specific chemogenetic manipulations during the formation (acquisition/consolidation) or retrieval of recognition memories. We consistently found that acute chemogenetic silencing of TMN^HA^ neurons disrupts the formation or retrieval of both social and object recognition memory in males and females. Conversely, acute chemogenetic activation of TMN^HA^ neurons during training or retrieval extended social memory in both sexes and object memory in a sex-specific fashion. These results suggest that the formation or retrieval of recognition memory requires the tonic activity of histaminergic neurons and strengthen the concept that boosting the brain histaminergic system can promote the retrieval of apparently lost memories.

## Introduction

In the mammalian brain, histaminergic neurons expressing histidine decarboxylase (*Hdc*, the catalyzing enzyme responsible for histamine biosynthesis) are located exclusively in the posterior hypothalamus, e.g., in the hypothalamic tuberomamillary nucleus (TMN^HA^), where they are densely packed in the ventral part and more loosely distributed in the proximity of the third ventricle and extend a diffuse network of fibres throughout the brain. Several lines of functional evidence indicate that this system regulates aspects of arousal, wakefulness^1–3^, feeding behaviour^4^ and motivation^5^. Genetic, pharmacological and electrophysiological manipulations have greatly contributed to the understanding of how the histaminergic system, by activating H_1_, H_2_ and H_3_ receptors in the brain, fine-tunes different behavioural responses. In recent years, designer receptors activated by designer drugs (DREADD) and optogenetic techniques have been used to control the activity of TMN^HA^ neurons, permitting unprecedented temporal, cellular and regional specificity. With these techniques, some authors have explored the complex functional arrangement of histaminergic circuits in the control of wakefulness and arousal^6–9^, eating behaviour^10^ and in the context of pathologies such as Parkinsonism^11^, maladaptive anxiety- and obsessive–compulsive-like behaviour s^12^. There is also convincing evidence that histamine (HA) is a regulator of consolidation and/or retrieval of different types of memory (reviewed in^13–15^. These studies used genetic models, local or systemic applications of histaminergic receptor ligands or HA synthesis and catabolism inhibitors, which allowed us to improve the temporal and anatomical dissection of the role of HA in short- and long-term memory. However, these approaches may suffer from caveats inherent to possible compensatory adaptation of genetic models, long-lasting effects of inhibitors or the impossibility of excluding stimulation or inhibition outside the TMN by ligands. As an example, H_3_R are expressed on non-histaminergic neurons, where their activation limits neurotransmitters and neuromodulators release. Moreover, these studies used only males. Although sex-dependent differences in animal cognition have been reported^16^, the effect of histaminergic system manipulation on memory processes in females remains to be characterized. Here, we evaluated the effect of chemogenetic inhibition or activation of TMN^HA^ neurons on long-term recognition memory formation or retrieval in males and females. For this purpose, we used two non-aversive and non-rewarded, spontaneous learning tasks using objects or conspecifics that may find their equivalent in humans when addressing episodic or declarative memory^17^. The behavioural tasks used here take advantage of the natural propensity of rodents to explore novelty in their environment, do not require punishments or food rewards, and allow for the dissection of the diverse phases of formation and retrieval of long -term memory.

## Results

### ***DREADD expression in TMN***^***HA***^*** neurons and ***ex vivo*** electrophysiology***

**Figure 1:**
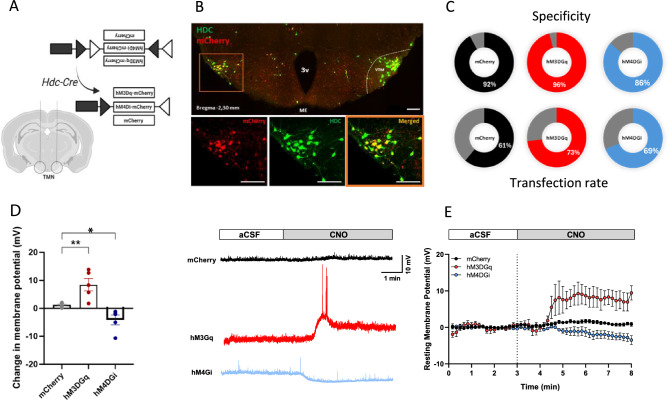
Chemogenetic targeting of the TMN. (**A**) Depiction of viral constructs targeted to the TMN of *Hdc*-Cre mice. (**B**) Representative images of hypothalamic sections double-labelled with antisera against Hdc and mCherry. (**C**) Pie chart representative of transfection rate (% Hdc^+^ neurons that are mCherry^+^); specificity (% mCherry^+^ neurons that are Hdc^+^) was hM4Gi = 86.4; hM3Gq = 96.7; mCherry = 92.1. (**D**) Bar plot showing changes in resting membrane potential in the opposite direction upon activation or inhibition of TMN^HA^ neurons. (**E**) Examples of current traces recorded from DREADD histaminergic neurons during the application of CNO. (**F**) CNO-induced depolarization of TMN^HA^ neurons was maintained throughout the application of CNO. N = 5–6 mCherry^+^ neurons from 2–3 mice per experimental group; ^*^P < 0.05 vs mCherry control; **P < 0.01 vs mCherry control.

Schematic drawing of viral constructs and site of injection area shown in Fig. 1A. Coronal sections of *Hdc*-Cre mice exhibited selective mCherry expression in *Hdc* immunopositive neurons in the posterior hypothalamus, mostly along the ventral edge of the TMN (Fig. 1B). Minimal extra-TMN expression was observed (specificity, hM3DGq = 98%; hM4DGi = 93%; mCherry = 94%), consistent with previous reports^6,9^. As shown in Fig. 1C, a substantial portion of *Hdc*-immunolabelled TMN^HA^ neurons expressed mCherry immunofluorescence in all three experimental groups of *Hdc*-Cre mice. To ascertain the effect of viral transfections, ex vivo whole-cell recordings were performed in DREADD-expressing histaminergic neurons of the ventral TMN identified by their mCherry fluorescence. Bath application of 5 µM CNO to hypothalamic slices expressing excitatory DREADD depolarized TMN^HA^ neurons and elicited action potentials (Fig. 1D–F); conversely, 15 µM CNO applied to hypothalamic slices expressing inhibitory DREADD hyperpolarized TMN^HA^ neurons. In hypothalamic slices of mice infused with the control viral construct AAV8-hSyn-DIO-mCherry, bath application of CNO was ineffective.

### ***Effect of chemogenetic inhibition of TMN***^***HA***^*** neurons in mice engaged in recognition memory tests***

#### *TMN*^*HA*^* neuron inhibition does not affect mouse sociability*

Social recognition memory is a fundamental behaviour to create and consolidate social groups. Rodents form transient memories of recently met individuals who may help them shape their behaviour in future encounters^18^. The paradigm used here provides a measure of sociability, which is the tendency of rodents to approach or avoid a conspecific. The estimation of mouse performance during the training phase consisted of determining their social motivation, which was quantified as the time mice spent exploring a social stimulus versus the empty cylinder. Impaired sociability may affect mouse preference for social novelty during memory retention, which is defined as the natural propensity to spend more time in the proximity of an unfamiliar mouse versus a familiar mouse. After the habituation session, the performance of AAV-hM4DGi and control AAV8-hSyn-DIO-mCherry (mCherry) mice was evaluated during the training phase to assess their sociability. All experimental and control male and female mice that received CNO before the sociability test spent significantly more time exploring the cup containing a sex-matched conspecific juvenile (Table 1). These results are consistent with data previously published in a similar context^19^.

**Table 1:** Exploration of the social and non-social stimulus during the sociability test. mCh = mCherry, control mice; Gi = hM4Gi DREADD. One-Sample Student t-test ****P < 0.0001.

			Time spent exploring (%)		
	AAV	Sex	Social	Non Social	*p*
Figure 2B	*mCh*	*Males*	66.7 ± 4.5	33.3 ± 4.5	****
	*Gi*	*Males*	72.4 ± 8.0	27.6 ± 8.0	****
Figure 2B	*mCh*	*Females*	67.0 ± 9.6	33.0 ± 9.6	****
	*Gi*	*Females*	67.8 ± 9.0	32.2 ± 9.0	****
Figure 2D	*mCh*	*Males*	69.8 ± 5.9	30.2 ± 5.9	****
	*Gi*	*Males*	64.7 ± 9.5	35.3 ± 9.5	****
Figure 2D	*mCh*	*Females*	70.3 ± 7.2	29.7 ± 7.2	****
	*Gi*	*Females*	63.0 ± 7.9	37.0 ± 7.9	****

### ***Chemogenetic silencing of TMN***^***HA***^*** neurons during memory formation or retrieval impairs long-term social recognition memory***

**Figure 2:**
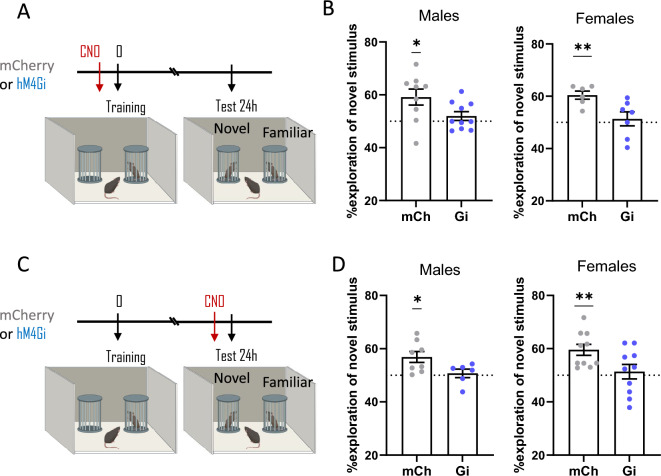
Chemogenetic inhibition of TMN^HA^ neurons deteriorates social memory. (**A**, **C**) Schematic drawings showing the sequence of the behavioural procedures and timing of CNO injections. Chemogenetic inhibition of TMN^HA^ neurons before acquisition/consolidation (**B**) or before retention (**D**) impaired social memory as tested 24 h after training. Shown are means ± S.E.M.s of 6–10 mice per experimental group; *P < 0.05; **P < 0.01.

To assess the effect of specific inhibition of TMN^HA^ neuron activity on social memory, the social discrimination test was performed 24 h after training, when male and female mice normally recognize the familiar juvenile and spend significantly more time exploring the novel conspecific. This was the case for mCherry control mice that received i.p. CNO injections either before the sociability test (Fig. 2A, B; one sample t test with theoretical mean = 50%: male mCherry, t_(8)_ = 3.0, *P* = 0.016; female mCherry, t_(5)_ = 6.7, *P* = 0.0011) or before the social recognition test (Fig. 2C,D; male mCherry, t_(7)_ = 3.3, *P* = 0.012; female mCherry, t_(9)_ = 4.5, *P* = 0.0014). When CNO was injected before the sociability test, inhibitory DREADD (hM4DGi) compromised the social memory of male (Fig. 2A,B; t_(9)_ = 1.2, *P* = 0.26) and female mice (t_(6)_ = 0.5, *P* = 0.63). The same effect was observed when the DREADD group received CNO before the social recognition test, as they did not discriminate between the two social stimuli (Fig. 2C, D; male mice, t_(5)_ = 0.4, *P* = 0.66; female mice, t_(9)_ = 0.5, *P* = 0.63).

We also performed a statistic comparison between groups, i.e. hM4DGi vs mCherry. Unpaired *t*-test analysis showed that both male and female hM4DGi mice receiving CNO before training performed significantly worse than mCherry mice (males, t_(19)_ = 2.15; *P* = 0.046; females, t_(13)_ = 2.79, *P* = 0.018). Similarly, the performance of male and female hM4DGi mice receiving CNO before testing was significantly lower than control mice (males, t_(14)_ = 2.24, *P* = 0.045; females, t_(20)_ = 2.39, *P* = 0.028).

### ***Chemogenetic silencing of TMN***^***HA***^*** neurons during memory formation or retrieval impairs long-term object recognition memory***

**Figure 3:**
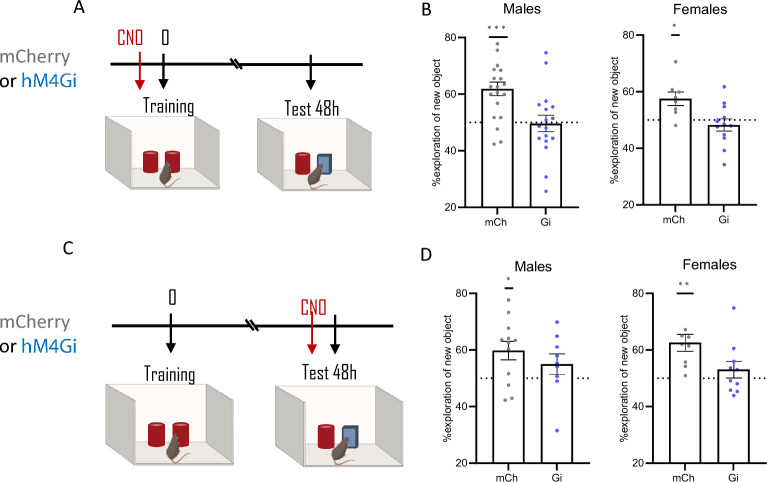
Chemogenetic inhibition of TMN^HA^ neurons deteriorates object recognition memory. (**A**, **C**) Schematic drawings showing the sequence of the behavioural procedures and timing of CNO injections. Chemogenetic inhibition of TMN^HA^ neurons before training (**B**) or before testing mice 48 h later (**D**) impaired recognition memory. Shown are means ± S.E.M.s of 9–20 mice per experimental group; *P < 0.05; **P < 0.01; ****P < 0.0001.

When exposed to a familiar object alongside a novel object, an experimental animal spends more time exploring the novel object than the familiar object. This behaviour is considered an indication that knowledge of the familiar object exists in the animal’s memory^20^. Figure 3 shows the effect of specific inhibition of TMN^HA^ neurons on novel object recognition memory. Control mCherry mice showed intact memory for the familiar object 48 h after training (Fig. 3B, D), as both males and females approached the novel object more than the familiar object when CNO was administered before training (one sample t test with theoretical mean = 50%: mCherry males t_(19)_ = 4.9, *P* = 0.0001; mCherry females t_(7)_ = 3.2, *P* = 0.016) or before the test at 48 h (mCherry males t_(11)_ = 3.0, *P* = 0.012; mCherry females t_(8)_ = 4.2, *P* = 0.003). The inhibitory DREADD hM4DGi compromised the memory of both male and female mice regardless of the timing of CNO injection, i.e., before training (G_i_ males, t_(16)_ = 0.1, *P* = 0.9; Gi females t_(11)_ = 0.8, *P* = 0.42) or testing (G_i_ males, t_(8)_ = 1.4, *P* = 0.21; G_i_ females, t_(9)_ = 1.1, *P* = 0.31). Group comparisons confirmed these results and showed that performance of both female and male hM4DGi mice receiving CNO before training were significantly lower than mCherry mice (unpaired *t*-test: males, t_(36)_ = 3.4, *P* = 0.0018; females, t_(18)_ = 2.9, *P* = 0.010; Fig. 3B). Similarly, hM4DGi-treated females, but not males, receiving CNO before the retrieval test performed significantly worse than control mice (males t_(19)_ = 1.0, *P* = 0.34; females, t_(17)_ = 2.3, *P* = 0.037; Fig. 3D).

## Effect of chemogenetic excitation of TMN^HA^ neurons of mice engaged in the recognition memory tests

### ***Chemogenetic activation of TMN***^***HA***^*** neurons during memory formation or retrieval improves long-term social recognition memory***

**Figure 4:**
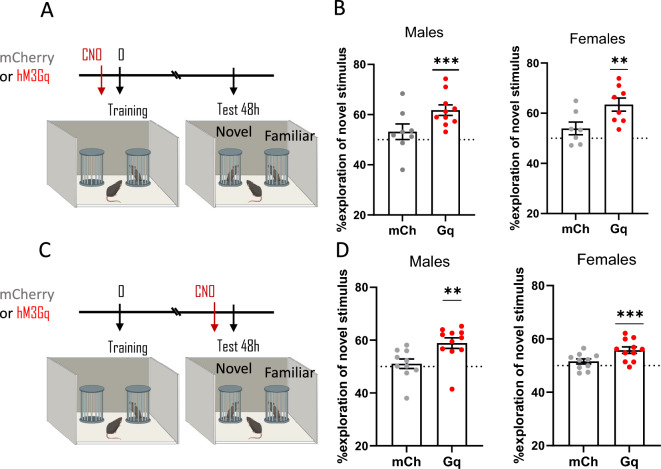
Chemogenetic activation of TMN^HA^ neurons ameliorates social memory. (**A**, **C**) Schematic drawings showing the sequence of the behavioural procedures and timing of CNO injections. Chemogenetic activation of TMN^HA^ neurons before acquisition/consolidation (**B**) or before retention (**D**) improved social memory as tested 48 h after training. Shown are means ± S.E.M.s of 7–11 mice per experimental group; **P < 0.01; ***P < 0.001.

We estimated the effect of chemogenetic activation of TMN^HA^ neurons on social recognition memory, which is normally forgotten 48 h after the sociability test session. Chemogenetic activation of TMN^HA^ neurons did not affect sociability, as male and female mice spent significantly more time exploring the cup containing a sex-matched conspecific juvenile with respect to the empty cup (Table 2 and Fig. 4A,C).

**Table 2:** Exploration of the social and non-social stimulus during the sociability test. mCh = mCherry, control mice; Gq = hM3Gq DREADD; One-Sample Student t-test *P < 0.05; ****P < 0.0001.

			Time spent exploring (%)		
	AAV	Sex	Social	Non Social	*p*
Figure 4B	*mCh*	*Males*	70.1 ± 8.2	29.9 ± 8.2	****
	*Gq*	*Males*	62.8 ± 11.3	37.2 ± 11.3	****
Figure 4B	*mCh*	*Females*	65.2 ± 8.9	34.8 ± 8.9	****
	*Gq*	*Females*	56.6 ± 10.6	43.4 ± 10.6	*
Figure 4D	*mCh*	*Males*	64.8 ± 8.6	35.2 ± 8.6	****
	*Gq*	*Males*	61.7 ± 12.3	38.3 ± 12.3	****
Figure 4D	*mCh*	*Females*	68.4 ± 10.1	31.6 ± 10.1	****
	*Gq*	*Females*	65.7 ± 9.2	34.3 ± 9.2	****

During the test performed 48 h after training, control mCherry mice displayed no social discrimination (Fig. 4B,D) when CNO was injected before training (one-sample t test with theoretical mean = 50%: mCherry male mice, t_(7)_ = 1.0, *P* = 0.34; mCherry female mice, t_(6)_ = 1.6, *P* = 0.17) or before testing (mCherry male mice, t_(9)_ = 0.6, *P* = 0.56; mCherry female mice, t_(9)_ = 1.6, *P* = 0.14), thus indicating that at this 48 h delay, social memory is forgotten. However, chemogenetic activation of TMN^HA^ neurons with CNO before training maintained social memory (Fig. 4B). Both male and female mice remembered the juveniles they previously encountered even 48 h after training and spent significantly more time exploring the new social stimulus than the familiar juvenile mouse (male mice t_(9)_ = 5.6, *P* = 0.0003; female mice, t_(7)_ = 5.1, *P* = 0.0013). Similarly, activation of TMN^HA^ neurons with CNO before the social recognition test rescued social memory, as illustrated in Fig. 4D (male mice, t_(10)_ = 4.454, *P* = 0.0012; female mice, t_(10)_ = 4.761, *P* = 0.0008).

When comparing the performance of hM3DGq vs mCherry mice, the unpaired *t*-test analysis showed that both male and female mice receiving CNO before training performed significantly better than mCherry mice (males, t_(18)_ = 2.37, *P* = 0.031; females, t_(15)_ = 2.59, *P* = 0.022). Male and female hM3DGq mice receiving CNO before the social recognition test as well showed better performances than control mice (males, t_(21)_ = 2.87, *P* = 0.099; females, t_(21)_ = 2.69, *P* = 0.014).

### ***Chemogenetic activation of TMN***^***HA***^*** neurons during memory formation or retrieval improves long-term object recognition memory in females only***

**Figure 5:**
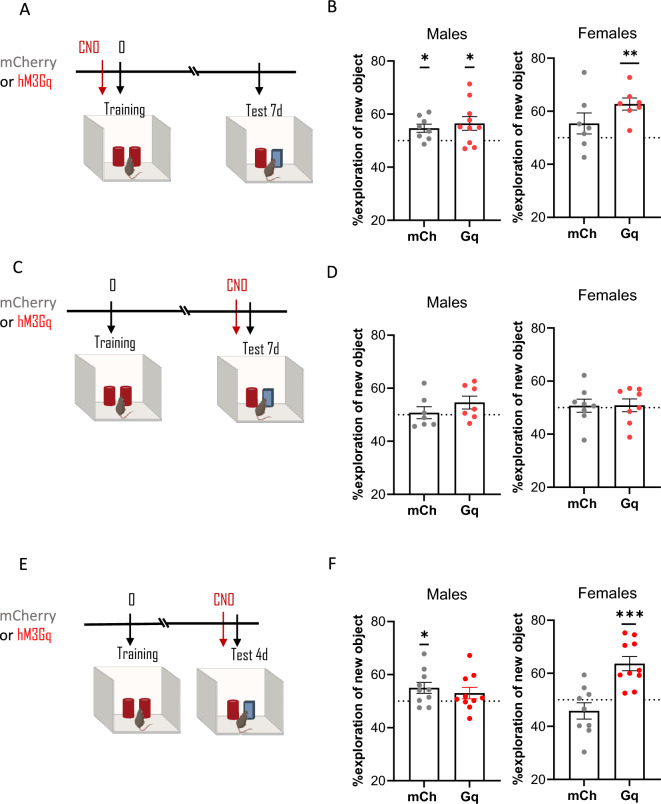
Chemogenetic activation of TMN^HA^ neurons ameliorates object recognition memory in a sex-dependent manner. (**A**, **C**, **E**) Schematic drawings showing the sequence of the behavioural procedures and timing of CNO injections. (**B**) Chemogenetic activation of TMN^HA^ neurons before acquisition/consolidation improved recognition memory of female mice as tested 7 days after training. (**D**) Chemogenetic activation of TMN^HA^ neurons before testing 7 days after training had no effect on recognition memory. (**F**) Chemogenetic activation of TMN^HA^ neurons before testing 4 days after training improved the recognition memory of female mice. Shown are means ± S.E.M.s of 7–17 mice per experimental group; *P < 0.05; **P < 0.01; ***P < 0.001.

To test the hypothesis that activation of histaminergic neurons is sufficient to evoke long-forgotten object memories, we evaluated the effect of TMN^HA^ neuron chemogenetic activation on object recognition memory 7 days after training. When CNO was injected before training (Fig. 5A), surprisingly, mCherry control male mice performed slightly better than chance (one-sample t test with theoretical mean = 50%: mCherry male mice, t_(7)_ = 3.1, *P* = 0.017), whereas the performance of mCherry control female mice was not different from chance (mCherry female mice, t_(5)_ = 0.8, *P* = 0.46; Fig. 5B). However, activation of TMN^HA^ neurons with CNO before training promoted object recognition memory in female mice at 7 days (t_(7)_ = 3.4, *P* = 0.011) but did not significantly affect male mice performance (t_(9)_ = 2.5, *P* = 0.031). Unexpectedly, the behaviour of Gq mice of either sex that received CNO before testing at 7 days (Fig. 5C) was not different from chance, similar to control mice (mCherry males t_(6)_ = 0.3, *P* = 0.75; Gq males t_(6)_ = 1.9, *P* = 0.11; mCherry females t_(7)_ = 0.3, *P* = 0.77; Gq females t_(7)_ = 0.4, *P* = 0.73; Fig. 5D). We speculated that reactivating TMN^HA^ neurons immediately before the test 7 days after training might be too late. Hence, we tested a new batch of mice 4 days after training and injected CNO before testing (Fig. 5E). mCherry control male mice performed above chance, exploring more the novel object (t_(9)_ = 2.5, *P* = 0.04), whereas female control mice showed no preference for the familiar object (t_(8)_ = 1.4; *P* = 0.2; Fig. 5F). Chemogenetic activation of TMN^HA^ neurons 4 days after training showed strong sexual dimorphism (Fig. 5F), as male mice were virtually unaffected (t_(9)_ = 1.4, *P* = 0.19), whereas all female mice preferred to explore the novel object (t_(9)_ = 5.1, *P* = 0.0007).

The results were corroborated by group comparisons using unpaired *t*-test. These analyses showed that object recognition performance of hM3DGq females, but not males, was better than mCherry mice when CNO was administered before training (males, t_(16)_ = 0.6, *P* = 0.58; females, t_(12)_ = 1.9, *P* = 0.077; Fig. 5E) or before the retrieval tests at 4 days (males t_(18)_ = 0.7, *P* = 0.52; females, t_(17)_ = 4.4, *P* = 0.0004; Fig. 5F) but not at 7 days (males t_(12)_ = 1.2, *P* = 0.27; females, t_(14)_ = 0.04, *P* = 0.97; Fig. 5D).

### ***Chemogenetic activation or inhibition of TMN***^***HA***^*** neurons does not affect short-term memory***

To assess whether our chemogenetic manipulations specifically affect long-term memory we evaluated their effects on short-term memory. CNO was administered before training and the social recognition test was performed 1 h after training (Supplementary Figure S1A). The protocol did not affect sociability, as experimental and control male and female mice spent significantly more time exploring the cup containing a sex-matched conspecific juvenile then the empty cup (Supplementary Table S1). During test, *Hdc*-Cre mice receiving intra-TMN injections of either hM3DGq or hM4DGi performed in a similar way to controls receiving mCherry showing a clear preference for the novel juvenile (one-sample t-tests with theoretical mean = 50%: mCherry males, t_(5)_ = 2.83, *P* = 0.037; Gq males, t_(5)_ = 4.29, *P* = 0.007; Gi males, t_(5)_ = 3.60, *P* = 0.015; mCherry females, t_(5)_ = 3.61, *P* = 0.015; Gq females, t_(5)_ = 3.74, *P* = 0.013; Gi females, t_(5)_ = 4.15, *P* = 0.009; one-way ANOVA: males F_(2, 15)_ = 0.06; *P* = 0.94; females F_(2, 15)_ = 0.17; *P* = 0.84; Supplementary Fig. S1B). Similarly, TMN^HA^ neurons activation or inhibition did not affect short-term memory in the novel object recognition test, as both males and females performed in the similar fashion to controls receiving mCherry showing a clear preference for the novel object (one-sample t-tests: mCherry males, t_(5)_ = 5.12, *P* = 0.004; Gq males, t_(5)_ = 2.85, *P* = 0.035; Gi males, t_(5)_ = 3.69, *P* = 0.014; mCherry females, t_(5)_ = 5.88, *P* = 0.002; Gq females, t_(5)_ = 5.14, *P* = 0.004; Gi females, t_(5)_ = 2.60, *P* = 0.048; one-way ANOVA: males F_(2, 15)_ = 1.80, *P* = 0.20; females F_(2, 15)_ = 0.79; *P* = 0.47; Supplementary Figure S1C-D). The absence of chemogenetic effects on social and object short-term memory also rules out the possibility that chemogenetic manipulation may affect non-mnemonic operations such as attention or sensory information processing.

## Discussion

In this study, we compared the performance of male and female mice in two non-aversive and non-rewarded memory tests, the social and object recognition test, when the brain histaminergic system is chemogenetically suppressed or enhanced. Mice express spontaneous long-term memory for conspecifics and objects for variable periods of time, and we and others previously demonstrated that pharmacologically or genetically histamine-deprived male mice show memory deficits in these tasks^19,21^;^22,23^. On the other hand, histaminergic ligands that enhance brain histamine release ameliorate memory retention in male rodents [see^14^ for a comprehensive review)].

By using a chemogenetic approach, we intended to avoid confounding elements inherent in testing genetically modified animals, where compensatory mechanisms may be engaged, or using histaminergic ligands that not only regulate histamine output but also the release of other neurotransmitters such as acetylcholine^24,25^ and dopamine^26^.

DREADD is a well-established technique used to selectively modulate the activity of specific neuronal populations in freely moving animals engaged in behavioural tasks. In the present study, the viral constructs were aimed at the TMN of *Hdc*-Cre mice. In coronal hypothalamic sections, mCherry immunofluorescence was found in the ventral edge of the TMN, consistent with the localization of histaminergic neurons in clusters E1 and E2^27^. The identity of labelled neurons was confirmed by the use of Hdc immunostaining. These results unequivocally demonstrated that selectively interfering with TMN^HA^ neuron activity has long-lasting effects on recognition memory. Indeed, when silencing TMN^HA^ neurons either before training or before testing, the memory of both male and female mice for conspecific or objects was compromised. These results are in agreement with previous studies using *Hdc*^-/-^ mice, which do not synthesize histamine or restrict histamine release by stimulating H_3_ receptors, both of which impair social memory^19,28^.

Other studies reported a strong effect of acute optogenetic silencing of TMN^HA^ neuron activity on wakefulness, promoting slow-wave sleep when mice enter their active nocturnal active period^6^. On the other hand, chemogenetic inactivation of TMN^HA^ neurons did not significantly affect locomotion in a familiar environment^9^. Although we did not assess arousal directly, Gi mice injected with CNO before training did neither change the exploration of objects or conspecifics during training, as assessed by normal sociability (Table 1), nor the short-term object or social memory ruling out chemogenetic effect on arousal, motivational, motoric or sensory processes.

In the social recognition task, 48 h after training, neither male nor female mice discriminated between familiar and unfamiliar mice. Therefore, we assessed whether activation of TMN^HA^ neurons would promote the retrieval of this forgotten memory in mice. The activation of TMN^HA^ neurons before training restored the ability to discriminate between familiar and unfamiliar conspecifics in male and female mice. It is conceivable that stimulating the histaminergic system during memory formation has long-lasting consequences on the expression of memory. More surprisingly, when the activity of TMN^HA^ neurons was enhanced before testing 48 h after training, social memory was also restored, suggesting that this memory was not completely lost but was more likely not accessible. A “helper”, such as boosting TMN^HA^ neurons, allows social memory to be retrieved and expressed.

Similar results were obtained in the object recognition test but only in females. Indeed, stimulating TMN^HA^ neurons during training promoted object recognition in females 7 days later. We found that pre-test activation of TMN^HA^ neurons 4 days,—but not 7 days, after training elicited memory recall of apparently lost object memories in female mice. On the other hand, the performance of control mCherry male mice was at variance, and TMN^HA^ neuron activation did not enhance their performance.

It is conceivable that latent memories for objects persist longer, up to 7 days post-training in female mice when the histaminergic system is activated during memory formation. However, acute activation of TMN^HA^ neurons immediately before testing is not sufficient for the expression of faded memories 7 days after training. Our protocol produced different results when compared to the results described by Nomura and coauthors^29^. They showed that acute systemic treatment with the H_3_ receptor antagonist thioperamide (which disinhibits histamine and other neurotransmitter release) before testing mice in the novel object recognition task induced the recall of forgotten memories up to 1 month after training. The retrieval-enhancing effect of thioperamide, though, was short-lasting, as mice did not discriminate between novel and familiar objects one day after treatment. Nonetheless, their study and our results indicate that memories persist even after they fade out with time and that by upregulating the histaminergic system, it is possible to retrieve lost memories.

Our object recognition results clearly indicate a sex-dependent effect of stimulation of TMN^HA^ neurons on memory. Sex differences in cognitive functions have been documented in humans^30^ and experimental animals^31,32^. For instance, robust sexual dimorphism was also described in the recruitment of circuits underlying fear memory and in the evolution of whole-brain networks over time^33^. In particular, fear learning and retrieval are mediated by distinct subsystems in the two sexes. Moreover, there is strong evidence in object recognition memory that females and males engage different brain circuits^34^. These observations and our results set the stage for further investigation to determine how TMN^HA^ pathways are engaged in male and female mice during recognition memory consolidation and retrieval.

Altogether, our results highlight the relevant role of the histaminergic system in the mechanisms responsible for recognition memory. In particular, using the specificity and selectivity of the chemogenetic approach, we confirmed the notion that activation of histaminergic neurons has the potential to retrieve forgotten memories^29^. Furthermore, our study uncovered a strong sexual dimorphism in memory recall that certainly warrants further investigation that may lead to relevant hypotheses for the treatment of cognitive disorders in humans.

## Methods

### Animals

For our study, we used a transgenic mouse line expressing Cre recombinase under the control of the *Hdc* promoter. The mouse strain was STOCK Tg(Hdc-cre)IM1Gsat/Mmucd, RRID:MMRRC_032079-UCD obtained from the Mutant Mouse Resource and Research Center (MMRRC) at University of California at Davis, an NIH-funded strain repository. The strain was donated to the MMRRC by Nathaniel Heintz, The Rockefeller University, GENSAT and Charles Gerfen, National Institutes of Health, National Institute of Mental Health.

Both hemizygous HDC-Cre male and female mice were used for these experiments. Animals were housed in humidity- and temperature-controlled rooms (22–24 °C) allowed free access to standard mouse chow and water and kept on a 12-h light/dark cycle (lights started at 7:00 a.m.). Breeding, housing, and all the experimental procedures were conducted in accordance with the Council Directive of the European Community (2010/63/EU) and the Italian Decreto Legislativo 26 (13 March 2014), approved by the Animal Care Committee of the University of Florence and Italian Ministry of Health and supervised by a veterinarian. Similarly, in France, all animal care and experimental procedures were in accordance with the INRAE Quality Reference System and with both French (Directive 87/148, Ministère de l’Agriculture et de la Pêche) and European legislation (Directive 86/609/EEC). They followed ethical protocols approved by the Region Aquitaine Veterinary Services (Direction Départementale de la Protection des Animaux, approval ID: B33-063-920) and by the animal ethics committee of Bordeaux CEEA50. The study is reported in accordance with ARRIVE guidelines.

Mice were handled for at least 4 days before experimental day 1 to allow them to acclimatize to human contact. All experiments were performed between 9:00 a.m. and 4:00 p.m.

#### Genotyping

In this study, we used inbred wild-type and histidine decarboxylase (*Hdc*)-Cre mice. The genotype was determined by using the PCR protocol developed by MMRRC at the University of California, Davis. (https://mmrrc.ucdavis.edu/protocols/032079Geno_Protocol.pdf).

#### Stereotaxic surgery and i.c.v. infusion procedure

Adult *Hdc*-Cre mice (8‒10 weeks old) were anaesthetized using a mixture of 100 mg/kg ketamine and 10 mg/kg xylazine and placed in a digital stereotaxic frame (Stoelting, Chicago, USA). The location of the TMN was determined bilaterally based on the atlas by Franklin and Paxinos (2007). A 500 nL volume of vector solution was infused bilaterally into the TMN (AP—2.4 mm, RL ± 0.6 mm, DV—5.0 mm from dura). The solution contained either the inhibitory AAV8-hSyn-DIO-hM4D(Gi)-mCherry (titre 4.4X10^12^ GC/mL catalog # 44,362) or the excitatory AAV8-hSyn-DIO-hM3D(Gq)-mCherry (titre 2.1X10^13^ GC/mL catalog # 44,361), both from Addgene (Cambridge, MA). The control groups received bilateral TMN infusion of the viral vector construct AAV8-hSyn-DIO-mCherry (titre 2.5X10^13^ GC/mL catalog # 50,459) (Adgene), which lacked engineered muscarinic receptors. The syringe was left in place for an additional 5 min for fluid diffusion, and then it was slowly withdrawn, and the scalp stapled to facilitate healing. Following surgery, mice were group housed with ad libitum access to food and water. The exogenous DREADD ligand clozapine-N-oxyde (CNO) was dissolved in 0.5% dimethyl sulfoxide (DMSO) and 99.5% sterile saline and delivered i.p. (1 mg/kg or 3 mg/kg for excitatory or inhibitory DREADD, respectively^35^) 45 min before training or before retrieval tests. The use of mCherry mice injected with CNO allows for controlling any off-target effect of CNO.

#### Novel object recognition test

Novel object recognition (NOR) relies on the motivational strength of novelty, as rodents search for and explore new objects with respect to familiar ones. The NOR paradigm consisted of two phases: training and testing. During training, two identical objects (cylinders or lego) were presented in a novel open field arena (40 × 40 × 40 cm, wood), and each mouse was left to explore them freely. Long-term memory was assessed at different times after training (1 h, 48 h, 4 or 7 days) by randomly replacing one of the objects with a novel object. The position of the objects (left/right) was randomized to prevent bias from order or place preference. In both phases, object exploration was defined as nose and whiskers pointed towards the object at a distance of less than 1–1.5 cm away (sitting on or turning around the objects was not considered exploratory behaviour), and an inclusion criterion of 20 s of total exploration was set at a 10-min exploration maximum for both objects, which reduces interindividual variability (Leger et al., 2013); otherwise, mice were excluded from analysis. Data are presented as the % of exploration of novel objects during the testing phase, calculated as time spent exploring the novel object/exploration time of both objects × 100.

#### Social discrimination test

The use of the social discrimination paradigm allows the evaluation of two critical but distinguishable aspects of social behaviour, namely, social preference, or sociability, and social recognition memory. Rodents form transient memories of recently met individuals who may help them shape their behaviour in future encounters^18^. The paradigm used here provides a measure of sociability, which is the tendency of rodents to approach or avoid a conspecific. The test was performed following a previously described protocol^19^. Briefly, the behaviour of 12- to 13-week-old adult mice was assessed in an open-field arena (45 × 25 cm and 20 cm high) in a sound attenuated room. Mice were habituated in the arena containing two empty wire cups on opposing sides for free exploration for 10 min. Twenty-four hours later, a 3–4-week-old mouse placed under one of the wire cups was used as a social stimulus for the experimental mouse, which was allowed to explore the juvenile for 10 min. The juvenile stimulus mice were habituated to remain under the wire cups for 30 min for several days before behavioural testing. After a variable time period, the experimental mouse was again placed in the arena containing the same stimulus animal under one wire cup and a novel unfamiliar juvenile mouse under the other cup for 10 min. The positions of the social stimuli were counterbalanced across subjects and trials to prevent bias due to place preference. Mice’ behaviour during all sessions was videotaped, and the time spent actively exploring the stimuli was analysed by an experienced observer unaware of the experimental groups. Exploration was defined as direct snout-to-cup contact, whereas the time spent climbing on the cups was disregarded. Data were expressed as the percentage of time spent exploring the social or non-social stimulus during the second session and the familiar or novel conspecific during the third session.

#### Histology

To verify the expression of viral vectors in TMN^HA^ neurons, mice were deeply anaesthetized with a mixture of ketamine/xylazine and transcardially perfused with phosphate-buffered saline (PBS) followed by 4% paraformaldehyde in PBS. Brains were harvested, postfixed in the same solution overnight at 4 °C and then cryoprotected in 30% sucrose in PB. Brains were sliced into 20 μm coronal sections using a freezing cryostat and processed for immunohistochemistry as previously described^36^. Briefly, sections were washed in PBS, blocked in 0.5% BSA/0.25% Triton X-100 in PBS for 1 h at room temperature and then incubated overnight at room temperature in blocking buffer containing the following primary antibodies: rabbit anti-HDC (American Research Products Inc., diluted 1/1000) and goat anti-mCherry (Sicgen, Cat. No. ab0040-200; diluted 1/1000). After 24 h, the sections were washed in PBS and incubated in blocking buffer containing the following secondary antibodies: donkey anti-rabbit (Abcam, Cambridge, UK; Cat. No. ab150073 Alexa Fluor 488; diluted 1/1000), and donkey anti-goat (Abcam, Cambridge, UK; Cat. No. ab150132 Alexa Fluor 594; diluted 1/1000) for 2 h. Sections were then washed in PBS mounted on slides, cover slipped and visualized on an Olympus BX63 microscope coupled to CellSens Dimension Imaging Software version 1.6 (Olympus, Milan, Italy). Images were captured with an Olympus XM 10 camera (Olympus, Milano, Italia). Specific bandpass filter sets for FITC and Texas Red were used to prevent bleed-through from one channel to the next. All images were processed and analysed using Fiji ImageJ (https://fiji.sc/). For quantification of labelled neurons, 4 sections of 3 mice per experimental group at bregma levels -1.94, -2.18, -2.03, -2.54 were chosen, cell counts were done manually and the total counts were: hM3DGq mice, mCherry^+^, *n* = 554; Hdc^+^, *n* = 725; mCherry^+^/Hdc^+^, *n* = 534. hM4DGi mice, mCherry^+^, *n* = 544; Hdc^+^, *n* = 672; mCherry^+^/Hdc^+^, *n* = 455; mCherry mice, mCherry^+^, *n* = 518; Hdc^+^, *n* = 735; mCherry^+^/Hdc^+^, *n* = 448. We calculated the transfection rate of viral constructs expression as (mCherry^+^Hdc^+^/Hdc^+^) and the specificity as (mCherry^+^Hdc^+^/mCherry^+^; Fig. 1C).

#### Preparation of hypothalamic slices and electrophysiological recordings

Hdc-Cre mice were deeply anaesthetized with isoflurane and decapitated for brain extraction. Coronal slices (250 μm) encompassing the ventral tuberomamillary nucleus (vTMN) were obtained with a vibroslicer (Leica VT1000S, Leica Microsystem, Wetzlar, Germany) in ice-cold cutting solution containing (in mM) NMDG (92), HEPES (20), glucose (25), NaHCO3 (30), NaH2PO4 (1.25), KCl (2.5), MgSO4 (10), and CaCl2 (0.5). Slices were allowed to recover for 1 h in warm (32–34 °C), low calcium artificial cerebrospinal fluid (aCSF) containing (in mM) NaCl (130), KCl (3.5), NaH_2_PO4 (1.25), NaHCO_3_ (25), glucose (10), CaCl_2_ (1) and MgSO_4_ (2). All solutions were equilibrated with a 95% O_2_ + 5% CO_2_ gas mixture. Slices were individually placed in the recording chamber of a Nikon Eclipse E600FN microscope equipped with 4X and 60X upright objectives, a CCD camera (Hamamatsu) for infrared videomicroscopy and a halogen lamp for fluorescence excitation. Slices were constantly perfused with warm (32–34 °C) carbo-oxygenated, high-calcium aCSF solution composed of (in mM) NaCl (130), KCl (3.5), NaH_2_PO_4_ (1.25), NaHCO_3_ (25), glucose (10), CaCl_2_ (2) and MgSO_4_ (1). Patch pipettes were made with a vertical puller (Narishige PP830, Narishige International Ltd, London, UK) from thin-walled borosilicate capillaries (Harvard Apparatus, London, UK) and back-filled with K-gluconate intracellular solution containing (in mM): 120 K-Gluconate, 15 KCl, 10 HEPES, 1 EGTA, 2 MgCl_2_, 5, Na2Phosphocreatine, 0.3 Na2GTP, and 4 MgATP (pH 7.3, 295–305 mOsm), resulting in a bath resistance of 3–4 MΩ. DREADD-expressing histaminergic neurons of the vTMN were identified by mCherry fluorescence. Whole-cell signals were sampled at 10 kHz and low-pass filtered at 3 kHz with an Axon Multiclamp 700B (Molecular Devices, Sunnyvale, CA, USA). To evoke the hM3DGq-mediated voltage response, 5 μM CNO was bath applied for five minutes after three minutes of stable baseline recording (Xiao y. Et al. 2015, 26,094,607). To elicit the hM4DGi-mediated response, the CNO concentration was raised to 15 μM based on our in vivo experiments. Traces showing a drift in membrane potential exceeding ± 5 mV were discarded. Voltage recordings were analysed with Clampfit 11.1 (Molecular Devices, Sunnyvale, CA, USA). The kinetics and magnitude of CNO-induced depolarization/hyperpolarization were analysed by dividing the traces into 10-s bins, while the effect of CNO on membrane potential was calculated as the ΔV of the 1-min average at the end of baseline and application.

### Statistical analysis

All behavioural data were analysed using Prism 6 (GraphPad Software, Inc.), and significant differences from chance (50%) for each group were determined using one-sample Student’s t tests for each group and between two groups (long-term memory) using unpaired t tests and between three groups (short-term memory) using one-way ANOVA. For electrophysiological data, normality was tested using the D’Agostino-Pearson test, and statistical significance was determined with a Mann‒Whitney U test. The level of statistical significance was set at *p* < 0.05 for all parameters. All data are presented as group mean values ± SEMs.

## Supplementary Information

Supplementary Information.

## Data Availability

All raw data are available upon request directed to the corresponding authors.
