# Supplementary material for: Chemogenetic activation or inhibition of histaminergic neurons bidirectionally modulates recognition memory formation and retrieval in male and female mice
Source: Sci Rep. 2024 May 17;14:11283. doi: 10.1038/s41598-024-61998-0 (PMC11101472; doi:10.1038/s41598-024-61998-0)
Supplement: Supplementary file 1 — Supplementary Information. [file 41598_2024_61998_MOESM1_ESM.docx]

**Chemogenetic activation or inhibition of histaminergic neurons bidirectionally modulates recognition memory formation and retrieval in male and female mice**

Alessia Costa^1^, Eva Ducourneau^2^, Lorenzo Curti^1^, Alessio Masi^1^, Guido Mannaioni^1^, Lola Hardt^2^, Essi F Biyong^2^, Mylène Potier^2^, Patrizio Blandina^1^, Pierre Trifilieff^2^, Gustavo Provensi^1^, Guillaume Ferreira^2^, M Beatrice Passani^3^.

^1^Dept. of Neuroscience, Psychology, Drug Research and Child Health, Pharmacology and Toxicology Unit, University of Florence, Italy.

^2^University of Bordeaux, INRAE, Bordeaux INP, Nutrition and Integrative Neurobiology, UMR 1286, 33077 Bordeaux, France.

^3^Department of Health Sciences, Clinical Pharmacology and Oncology Unit, University of Florence, Italy.

**Supplementary Material**

**Supplementary Figure S1**. **CNO application before training did not affect recognition memory 1 hour after training in hM4DGi and hM3DGq expressing mice. (A) Schematic drawing showing the sequence of the behavioural procedures and timing of CNO injections in the social recognition task. B) Cognitive performance of hM3DGq (Gq), hM4DGi (Gi) or mCherry (mCh) expressing male and female mice in the social recognition task C) Schematic drawings showing the sequence of the behavioural procedures and timing of CNO injections in the novel object recognition task. D) Cognitive performance of hM3DGq (Gq), hM4DGi (Gi) or mCherry (mCh) expressing male and female mice in the novel object recognition task. Shown are means ± S.E.M.s of 6 mice per experimental group; *P<0.05, **P<0.01.**

|  |  |  | **Time Spent Exploring** | | |  |  |  |  |  |
| --- | --- | --- | --- | --- | --- | --- | --- | --- | --- | --- |
|  | **AAV** | **Sex** | **Social**  **(%)** | **P**  **P** | **Total Investigation Time (s)** | | | **Sociability Index (SI)** |  |  |
| Suppl Fig 1 | *mCh* | *Males* | 60.15 ± 7.20 | * | 195.27 ± 59.02 | | | 0.12 ± 0.09 |  | ns |
|  | *Gq* | *Males* | 72.04 ± 13.84 | * | 217.38 ± 49.43 | | | 0.23 ± 0.18 |  |  |
|  | *Gi* | *Males* | 65.13 ±12.98 | * | 159.58 ± 49.15 | | | 0.17 ± 0.13 |  |  |
| Suppl Fig 1 | *mCh* | *Females* | 64.49 ± 7.91 | ** | 191.88 ± 41.98 | | | 0.15 ± 0.09 |  | ns |
|  | *Gq* | *Females* | 62.07 ± 6.69 | ** | 132.26 ± 76.71 | | | 0.27 ± 0.18 |  |  |
|  | *Gi* | *Females* | 66.63 ± 6.84 | ** | 116.31 ± 33.40 | | | 0.29 ± 0.12 |  |  |

**Suppl. Table 1** Exploration during the sociability test. mCh = mCherry, control mice; Gq = hM3DGq;

One-Sample Student t-test *P < 0.05;**P<0.01; ***P<0.001; ****P < 0.0001 social stimulus vs 50%;

One-way ANOVA: ns, non-significant.
